# A Versatile, Portable Intravital Microscopy Platform for Studying Beta-cell Biology *In Vivo*

**DOI:** 10.1038/s41598-019-44777-0

**Published:** 2019-06-11

**Authors:** Christopher A. Reissaus, Annie R. Piñeros, Ashley N. Twigg, Kara S. Orr, Abass M. Conteh, Michelle M. Martinez, Malgorzata M. Kamocka, Richard N. Day, Sarah A. Tersey, Raghavendra G. Mirmira, Kenneth W. Dunn, Amelia K. Linnemann

**Affiliations:** 1 Department of Pediatrics, Indiana University School of Medicine, Indianapolis, IN USA; 2 Herman B Wells Center for Pediatric Research, Indianapolis, IN USA; 3 The Center for Diabetes and Metabolic Diseases, Indiana University School of Medicine, Indianapolis, IN USA; 4 Department of Biochemistry and Molecular Biology, Indiana University School of Medicine, Indianapolis, IN USA; 5 Department of Medicine, Division of Nephrology, Indiana University School of Medicine, Indianapolis, IN USA; 6 Department of Cellular and Integrative Physiology, Indiana University School of Medicine, Indianapolis, IN USA

**Keywords:** Biological models, Endocrine system and metabolic diseases

## Abstract

The pancreatic islet is a complex micro-organ containing numerous cell types, including endocrine, immune, and endothelial cells. The communication of these systems is lost upon isolation of the islets, and therefore the pathogenesis of diabetes can only be fully understood by studying this organized, multicellular environment *in vivo*. We have developed several adaptable tools to create a versatile platform to interrogate β-cell function *in vivo*. Specifically, we developed β-cell-selective virally-encoded fluorescent protein biosensors that can be rapidly and easily introduced into any mouse. We then coupled the use of these biosensors with intravital microscopy, a powerful tool that can be used to collect cellular and subcellular data from living tissues. Together, these approaches allowed the observation of *in vivo* β-cell-specific ROS dynamics using the Grx1-roGFP2 biosensor and calcium signaling using the GcAMP6s biosensor. Next, we utilized abdominal imaging windows (AIW) to extend our *in vivo* observations beyond single-point terminal measurements to collect longitudinal physiological and biosensor data through repeated imaging of the same mice over time. This platform represents a significant advancement in our ability to study β-cell structure and signaling *in vivo*, and its portability for use in virtually any mouse model will enable meaningful studies of β-cell physiology in the endogenous islet niche.

## Introduction

All forms of diabetes are associated with failure of the insulin-producing pancreatic islet β-cells. The islet is a complex micro-organ consisting of multiple cell types, including neuro-endocrine, endothelial, neuronal, and immune cells, that integrate both local and systemic signals to tightly regulate hormone secretion. Therefore, understanding a complex disease like diabetes requires a complete understanding of the cell biology of islets. Whereas isolated islets have been used for decades in studies *in vitro* that have established the foundations of islet biology, systemic inputs and interactions that are critical to *in vivo* function are lost when islets are removed from the pancreas. The incomplete *in vitro* environment is a particular deficiency in the context of a disease like diabetes, whose progression stems from changes in islet cell function occurring in a complex and continuously changing biochemical and immunological milieu^1,2^.

One approach that has been widely used to study cell biology *in vivo*, particularly in the fields of neurobiology and cancer research, is intravital microscopy (IVM). The development of multiphoton microscopy has made it possible to collect images with subcellular resolution deep in living tissues. In the realm of islet biology, IVM of the pancreas has been used to characterize alterations in immune cell behavior in diabetes, and physiological regulation of microvascular flow^3–8^. IVM of islets transplanted under the kidney capsule or into the anterior chamber of the eye has been used to characterize islet tissue development^9^ and revascularization^10^, respectively. When combined with fluorescent biosensors, IVM has the added capability to characterize specific biochemical pathways in individual cells *in vivo*, providing unique insights into intracellular signaling, cell physiology and pathophysiology in the most relevant context of the intact organism^10^. However, leveraging the power of this approach can be challenging because it depends upon expression of the fluorescent biosensor in the cell-type of interest.

The classical approach to biosensor expression has been to generate a transgenic mouse line that selectively expresses biosensor in the cell type of interest. When successful, this approach can provide a valuable method to study cellular function *in vivo*. However, several drawbacks to this approach exist that can limit versatility. For example, this process requires breeding strategies which can be time-consuming and complicated if the biosensor line needs to be combined with a second transgene or knockout model. In addition, since any given biosensor mouse typically provides a read-out for a single analyte and is restricted to currently available mouse lines, new lines of inquiry into other pathways could require the generation of additional transgenic lines. To circumvent these issues, we developed a platform for selectively expressing biosensors in β-cells *in situ* using adeno-associated virus serotype 8 (AAV8) mediated gene transfer. This platform provides the flexibility to transition rapidly between any currently available or newly designed biosensor under control of the promoter of choice and any animal model system. Here, we demonstrate that we can achieve β-cell specific expression of 2 different biosensors, ROS-sensitive roGFP2 or calcium-sensitive GcAMP6s, using an insulin promoter-driven AAV8 system. We also show that the insulin promoter-driven construct can be moved to an adenoviral system for *in vivo* biosensor-based studies of transplanted islets.

Our proof-of-principle studies with both the roGFP2 and GcAMP6s biosensors also demonstrate that these viral expression approaches can be combined with either confocal or two-photon microscopy to monitor β-cell function and signaling in both the endogenous pancreas and in islets transplanted under the kidney capsule. Lastly, we present specialized methods of surgery and organ presentation necessary for high-resolution IVM of islets, using either single-point or longitudinal studies over the course of weeks, using an abdominal imaging window.

## Results

### Intravital microscopy of endogenous islets

**Figure 1:**
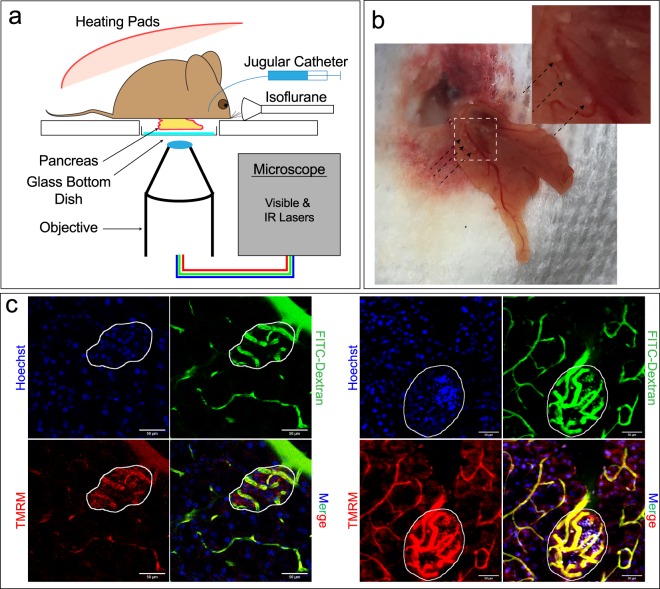
Intravital Microscopy of Endogenous Islets. (**a**) Schematic depiction of the setup for intravital microscopy of the mouse pancreas. (**b**) Widefield view of the pancreas, as seen through a coverslip-bottomed dish. The islets within the pancreas are highlighted by black arrows. (**c**) Mice were injected with Hoechst (blue) to label nuclei, a 150 k Da FITC-conjugated dextran (green) to label vasculature, and TMRM (TMRM) to label mitochondria. Both a single plane image (left) and a projection of a 3D image volume (right) collected from the pancreas shows the increased nuclei count, vascularity, and mitochondrial activity of islets (outlined in white) compared to surrounding exocrine tissue.

For IVM of a mouse pancreas, we used the approach shown in Fig. 1a. Briefly, the pancreas is surgically exposed (as described in “Methods”) and placed on the glass window of a cover-slip-bottomed dish, which is then mounted on the stage of an inverted microscope. Animal body temperature is maintained through the use of heating pads placed on the stage, a warming blanket placed over the animal, and an objective heater, while core temperature is monitored via a rectal probe. Anesthesia is accomplished using isoflurane, administered through a scavenging nosepiece. A jugular catheter is implanted to support intravenous (IV) injection of probes and/or drugs.

High resolution IVM requires that motion imparted by respiration and heartbeat is reduced to submicron levels. In our preparation, the pancreas is largely stabilized by the force of the animal’s body weight against the cover-slip, with additional stability provided by gauze placed between the exposed organ and abdominal wall. When the pancreas is resting against the imaging dish, islets are visible to the naked eye (Fig. 1b, black arrows). Superficial islets can often be imaged using confocal microscopy, however multiphoton microscopy can be used to extend the depth of imaging *in vivo*, which in the case of the pancreas extends to ~100 microns. We find that this reach typically gives us optical access to 2–5 islets per pancreas, with majority of the islets appearing the body region of the pancreas. Figure 1c shows two typical examples, one of a single plane and one of a projected 3D volume, of islets in the pancreas following IV injection of Hoechst (to label nuclei), a 150 kDa fluorescein-conjugated dextran (to label vasculature), and tetramethylrhodamine (TMRM, a probe that accumulates in active mitochondria). With these probes, islets are easily distinguished by their characteristically higher density of nuclei and vasculature, and by strong TMRM labeling, reflecting their high metabolic activity.

### AAV8-mediated expression of fluorescent protein biosensors as a flexible tool for studying β-cell biology in the pancreas *in vivo*

**Figure 2:**
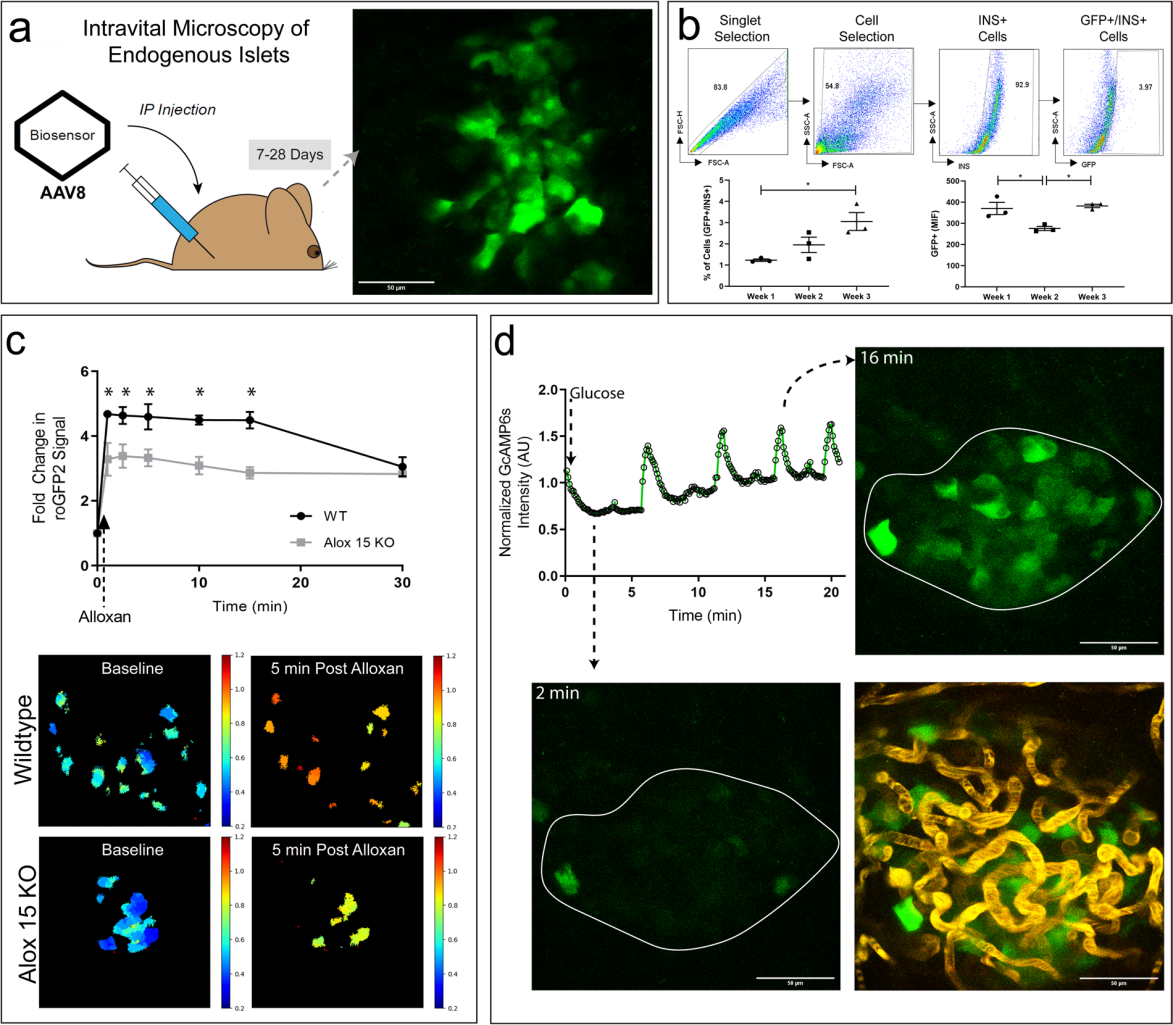
AAV8 Expression of Fluorescent Protein Biosensors as a Flexible Tool for Studying β-Cell Biology in the Pancreas *in vivo*. (**a**) Schematic depiction of pancreas IVM using AAV8 to label islets *in vivo*. Virus is injected IP 7–28 days prior to IVM. A representative projected image volume collected from the pancreas of a mouse 3 weeks after IP injection of AAV8-Grx1-roGFP2 is shown. (**b**) Dispersed cells from islets expressing the Grx1-roGFP2 biosensor were stained with anti-insulin and GFP^+^ INS^+^ population was evaluated by flow cytometry. Gating strategy (top) and quantification (bottom) of GFP^+^ INS^+^ cells are shown. N = 3. Data presented as mean +/− SEM (*p-value < 0.05). (**c**) Top: Fold change over baseline in Grx1-roGFP2 ratio from β-cells *in vivo* in the pancreata of WT (black) and Alox15^−/−^ (grey) mice over 30 minutes post IV injection of alloxan. Bottom: Representative ratiometric images of Grx1-roGPF2 changes in β-cells of WT and Alox15^−/−^ mice within the endogenous pancreas at 5 minutes after IV injection of 85 mg/kg alloxan monohydrate. Data are means ± SEM (N = 3, p < 0.05). (**d**) Top left: GcAMP6s intensity measures collected *in vivo* from an entire islet (white outline) over time after a 1 g/kg IP glucose bolus. Representative images of low and high GcAMP6s intensity are shown. Bottom right: A 70 k Da Texas red dextran was administered IV to label the vasculature of the islet.

To exploit the potential of fluorescent protein biosensors for studies of β-cell function *in vivo* without the need for creation of transgenic mice, we developed an AAV8 to package β-cell-selective biosensors using a hybrid insulin/rabbit β-globin promoter^11^. RoGFP is a redox-sensitive GFP-based probe that is able to report changes in intracellular reactive oxygen species (ROS) through the ratio of emissions upon excitation at 405 nm and 488 nm^12,13^ or at 800 nm and 900 nm^14,15^. The ratiometric properties of roGFP allows for measurements to be collected independent of the amount of roGFP expressed or the optical pathlength^16^. Here, we used roGFP2 containing human glutaredoxin-1 (Grx1-roGFP2), which is sensitive to the glutathione redox cycle in the cytoplasm, but is resistant to errors in measurement directly related to photobleaching^17^. Since AAV8 has tropism for the pancreas when administered via intraperitoneal (IP) injection^18^, the custom biosensor constructs are rapidly expressed specifically in endogenous β-cells at levels sufficient for detection by IVM (Figs 2a and S1).

To measure the timing of AAV8-mediated expression of fluorescent biosensors in the pancreas, we isolated islets from mice 1–3 weeks following IP administration of ~2 × 10^11^ genome copies of AAV8 containing Grx1-roGFP2 and used flow cytometry to quantify the cells positive for GFP and immunofluorescently labeled for insulin. As shown in Fig. 1c, we observed a steady increase in the percentage of β-cells that express Grx1-roGFP2 over time, with 1.23% of β-cells expressing biosensor at week 1 increasing to 3.05% at week 3. The mean intensity of fluorescence of the GFP-positive cells remained relatively constant over time.

To demonstrate the portability of our AAV8 viral expression system into additional mouse models, we applied it to studies of ROS in a mouse model that would confer altered antioxidant response compared to wild-type (WT) mice. *Alox15* knockout mice (*Alox15*^−/−^) lack the 12/15-lipoxgenase enzyme, which we previously reported to confer protection against oxidative stress in β-cells^19^. We hypothesized that this animal model would therefore exhibit altered kinetics of ROS dynamics in the β-cell that would correlate with changes in oxidative damage compared to WT controls. *Alox15*^−/−^ and WT littermates were each administered a single IP injection of AAV8-INS-Grx1-roGFP2 three weeks prior to intravital imaging to allow for biosensor expression. Superficial islets were first located using confocal microscopy, and then repeatedly imaged at 405 nm and 488 nm excitation over time after IV infusion of 80 mg/kg alloxan. The ratio of emissions excited at 405 nm and 488 nm were then calculated. Figure 2c shows that alloxan induced a rapid and extended release of ROS in β-cells of WT mice, as reflected in a 4.6-fold increase compared to baseline in the standardized 405/488 intensity ratio. In contrast, the response of β-cells in the *Alox15*^−/−^ mice was attenuated by 30%, consistent with our previous studies^19^. The behavior of Grx1-roGFP2 *in vivo* can also be observed in isolated islets infected with the adenoviral version of the construct *in vitro* (Fig. S2). A stronger response can be seen *in vivo* compared to *in vitro* conditions, which may reflect differences in timing or effective dose of biosensor delivered *in vivo*.

To demonstrate the flexibility of our viral system, we substituted a second biosensor, calcium sensitive GcAMP6s, for Grx-1-roGFP2 to create AAV8-INS-GcAMP6s. GcAMP6s is a widely-used calcium sensor that has been well-characterized in many cell types, both *in vitro* and *in vivo*^20^. For imaging of calcium dynamics *in vivo*, a 9-week-old wild-type (WT) C57Bl/6 J mouse was administered a single IP injection of AAV8-INS-GcAMP6s three weeks prior to IVM to allow for biosensor expression. An individual islet was identified using 920 nm excitation of GCamp6s then imaged over time after IP injection of a glucose bolus (1 g/kg) (Fig. 2d). Consistent with *in vitro* studies^21^, we observed synchronized, 2-fold changes in GCamp6s fluorescence across all the β-cells of the imaged islet *in vivo* (Supplemental Video, and quantified in Fig. 2d). The synchronicity of the β-cells leads to discrete peaks in the intensity trace from the islet and observable oscillations over time. A fluorescent red dextran was injected to highlight the vasculature of the oscillating islet.

### Adenovirus-mediated expression of fluorescent protein biosensors as a flexible tool for studying the cell biology of transplanted islets *in vivo*

**Figure 3:**
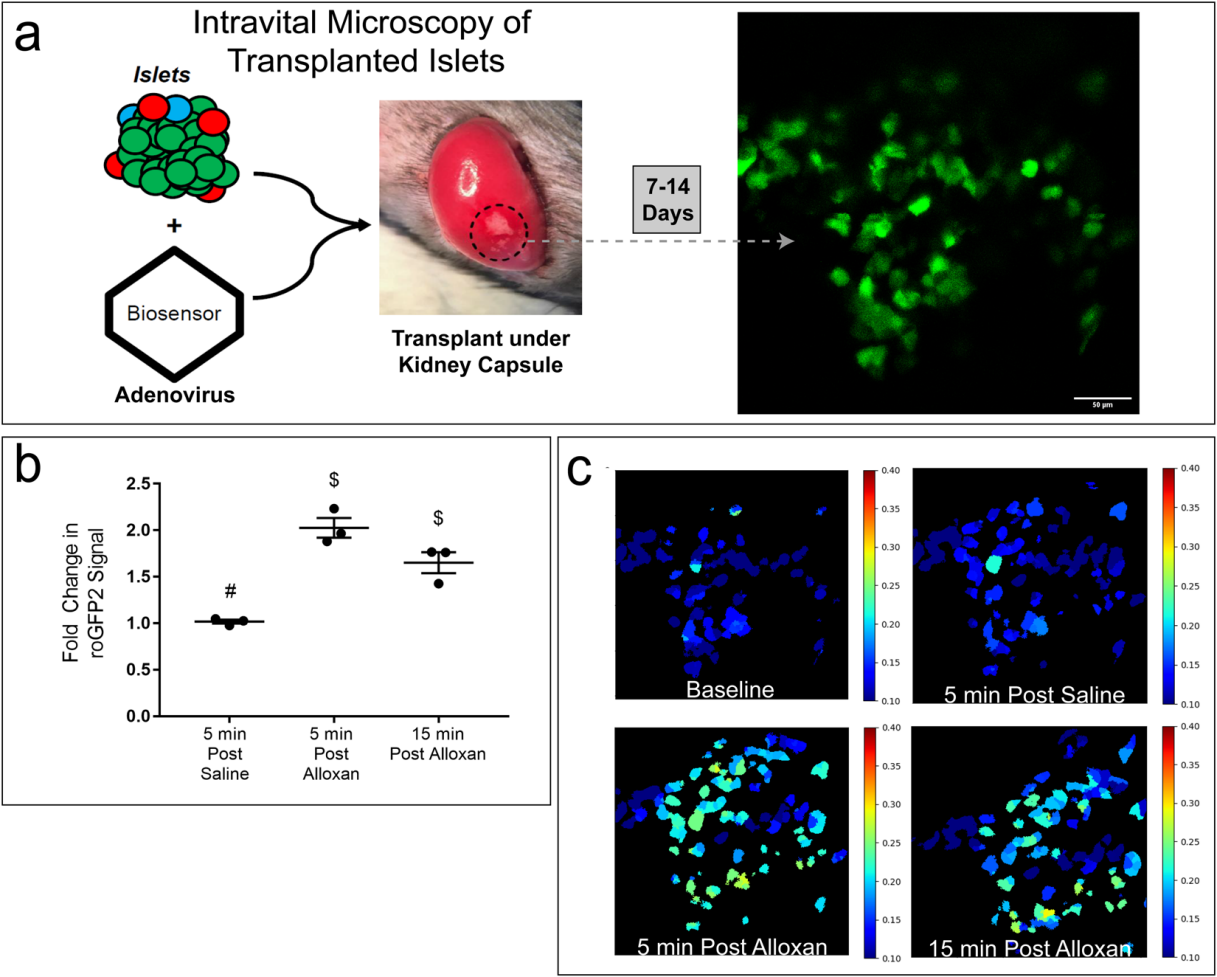
Adenovirus Expression of Fluorescent Protein Biosensors in Islets Transplanted under the Mouse Kidney Capsule. (**a**) Top left: Schematic depiction of IVM of biosensor-labelled isolated primary mouse islets transplanted under the mouse kidney capsule after adenoviral transduction *in vitro*. Right: Representative image of an islet graft under the kidney capsule labeled with Grx1-roGFP2. (**b**) Fold change over baseline in β-cell-specific Grx1-roGFP2 from 3 mice, 5 minutes after IV saline injection, and 5 and 15 minutes after IV injection of 80 mg/kg alloxan monohydrate. Pairwise t-tests were calculated for each time point between groups. Data are means ± SEM (N = 3, *p < 0.05). (**c**) Representative 405/488 ratiometric images of Grx1-roGPF2 changes in β-cells within the islet transplant under the kidney capsule at baseline, 5 minutes after IV saline injection, and 5 and 15 minutes after IV injection of 80 mg/kg alloxan monohydrate.

To reestablish an *in vivo* environment for isolated tissue, islets can be transplanted into several locations within a recipient mouse. For IVM studies, the anterior chamber of the eye^22,23^ and under the kidney capsule^9^ are the primary reported locations for transplantation. Isolated islet transplantation under the kidney capsule is a well-characterized model in which islets are engrafted into a host tissue, where they revascularize over the subsequent period of days to weeks^24,25^. Despite the islet not being in its native environment, this technique provides several key advantages, including utilization of novel host/donor combinations (i.e., human islets) and interrogation of novel transplantation methods (i.e., islet encapsulation). We utilized a syngeneic transplantation model, in which C57Bl/6J mouse islets transduced with Ad-INS-Grx1-roGFP2 *in vitro* are transplanted under the kidney capsule of recipient C57Bl/6J mice (Fig. 3a). An example of an image collected from roGFP2-expressing islets 10 days after transplantation into the mouse kidney is shown. Figure 3b shows representative results of a study in which an individual islet was identified and then repeatedly imaged by confocal microscopy following IV injection of saline, followed by IV injection of 80 mg/kg alloxan monohydrate. Whereas infusion of saline alone had no effect on the 405/488 roGFP2 excitation ratio, infusion of alloxan resulted in a significant and highly reproducible doubling of the roGFP2 ratio within 5 minutes. The ratio then decreased within 15 minutes, presumably due to activation of the endogenous antioxidant response and mitigation of intracellular ROS within the transplanted islets (Fig. 3c).

### Longitudinal intravital studies of β-cell biology using an abdominal imaging window

**Figure 4:**
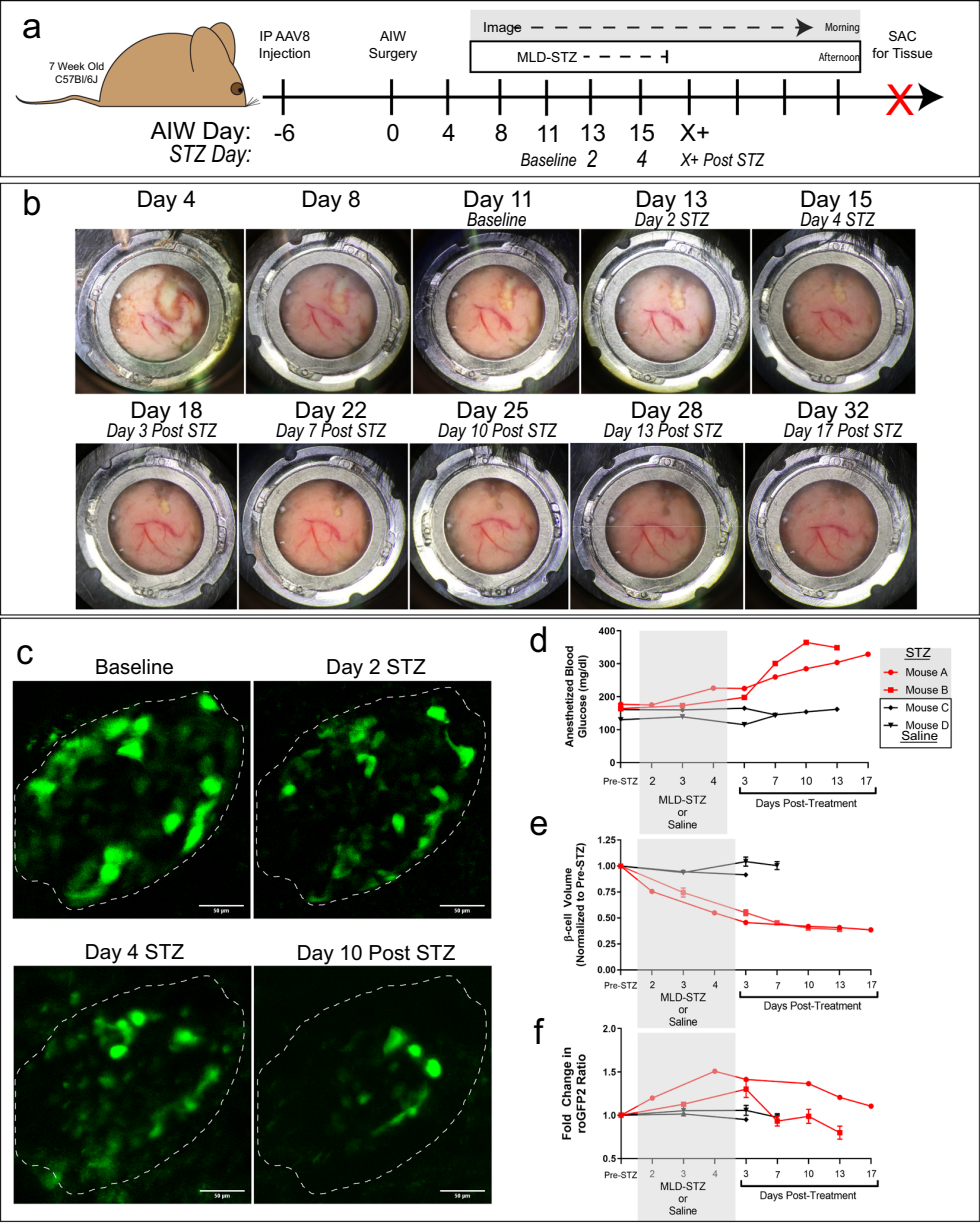
Longitudinal intravital studies of β-cell biology using an abdominal imaging window. (**a**) Schematic of AAV8-INS-Grx1-roGFP2, abdominal imaging window (AIW), and multi-low-dose streptozotocin (MLD-STZ; 55 mg/kg/day) experiment. Intraperitoneal injection of AAV8-packaged biosensor occurred 6 days prior to AIW surgery (day 0). Baseline images were collected in the morning of day 11 and STZ started in the afternoon of day 11. STZ continued for 5 days while imaging continued until the window integrity was compromised at Day 32. The pancreas was recovered and fixed for endpoint analyses. (**b)** Representative widefield images of the pancreas as seen through the AIW over time. Above each image are both the number of days post AIW implantation and the time point of the STZ challenge. (**c**) Representative projected images of the Grx1-roGFP2-labeled β-cell volume at baseline (Day 11), day 2 of STZ, day 4 of STZ, and day 10 post-STZ. The white dotted line indicates the perimeter of the Grx1-roGFP2-labeled islet volume at baseline. (**d**) Blood glucose readings collected 30 minutes after the start of anesthesia for each imaging session for STZ (red) and saline (black) treated mice. (**e**) Islet volume calculated for STZ (red) and saline (black) treated mice before, during, and after MLD-STZ challenge. (**f**) Grx1-roGFP2 ratiometric data collected from labeled β-cells using 800/900 nm 2-photon excitation before, during, and after in STZ (red) and saline (black) treated mice. For e and f, while the single line is representative of a mouse, 1–4 islets were imaged per mouse.

To capture chronic, longitudinal changes in ROS, we implanted a modified version of a previously described abdominal imaging window (AIW) into C57Bl/6J mice^26^ (modifications are fully described in Methods). AIWs provide a way to image the endogenous tissue on the time scale of days to weeks^26^, allowing stable imaging of the same islets over time. We implanted AIWs in AAV8-INS-Grx1-roGFP2-injected WT mice. Mice were then injected daily over the course of 5 days with 55 mg/kg streptozotocin (multiple low-dose or MLD-STZ challenge) (Fig. 4a). The MLD-STZ challenge is a well-characterized pharmacological model used to induce diabetes, where hyperglycemia develops within days after the last STZ injection, and is associated with islet infiltration and reduced β-cell mass due to apoptosis^27,28^. Images of the window preparation collected over time demonstrated that stable positioning of the pancreas was obtained within ~8 days of window placement and was maintained for an additional 24 days (Fig. 4b). Second harmonic generation images of collagen were used to verify that we returned to the same islet during each imaging session (Fig. S3). Figure 4c shows mean intensity projections of multiphoton fluorescence image volumes of Grx1-roGFP2-labeled β-cells within an islet in the pancreas of a living mouse collected at baseline (11 days after window implantation, prior to beginning the MLD-STZ challenge), at days 2 and 4 of STZ treatment, and 10 days after MLD-STZ treatment. These images demonstrate that the islet volume and architecture changes dramatically in response to MLD-STZ.

Consistent with previous reports^27,28^, MLD-STZ resulted in elevated blood glucose levels within 3 days of treatment and overt hyperglycemia was observed by 17 days post treatment (Fig. 4d). We measured the GFP positive volume from each islet (Fig. S4) and observed a rapid ~50% reduction in the β-cell volume by the end of STZ treatment, which then stabilized at ~20% of the initial volume by 10-days post-treatment (Fig. 4e). The reduced β-cell volume and increased blood glucose levels after MLD-STZ are indicative of β-cell ablation.

To characterize changes in ROS occurring over time after MLD-STZ, we used an approach in which the ratio of two-photon stimulated fluorescence of Grx1-roGFP2 excited at 800 nm to that excited at 900 nm is used to assay ROS^14,15^. Although this approach provides less dynamic range than the 405/488 excitation ratio used in confocal microscopy (Fig. S2e), two photon excitation penetrates deeper into tissue, supporting analyses from islets deeper in the pancreas^15^ and allowing for more data to be gathered from multiple islets. Grx1-roGFP2 ratios measured from the imaged β-cells showed that biosensor ratio peaked near the end of STZ administration (Fig. 4f). This effect subsided ~13 days after MLD-STZ occurred in conjunction with the stabilization of β-cell volume. The somewhat muted response of the roGFP2 sensor may reflect the fact that measurements were obtained from the subpopulation of cells that have been able to resist STZ ablation, thus underestimating the magnitude of the ROS response. One technical limitation of our methods is that the stability of the window and the imaging depth of the islets dictate if a mouse can be imaged longitudinally. In our cohort of animals, the saline-treated mice were removed from our study at earlier time points because islets from these mice were unable to be continually imaged. Regardless of specific imaging parameters or islet imaging depth, overall stability of the window for the duration of the study still allows for pancreas recovery at the end of AIW-IVM studies for additional analyses.

## Discussion

IVM is a valuable tool to evaluate a number of different physiological functions (e.g., microvascular flow, mitochondrial function, transport) using fluorescent probes introduced either intravenously or intraperitoneally^8,29^. While studies examining transplanted islet function have been informative^22^, examples of IVM for the study of endogenous β-cell function and signaling *in vivo* are sparse^4^, likely due to the historical need for transgenic animals expressing fluorescent biosensors. Thus, the array of measurable physiological functions can be greatly expanded through the use of virally-packaged fluorescent protein biosensors engineered to quantitatively measure a wide variety of physiological and cellular functions without the need to create multiple transgenic mouse lines. Here, we demonstrate an IVM platform that supports high-resolution microscopy of islet cells *in vivo*, either acutely or in longitudinal studies that, when combined with a virus-based delivery system, can be used to characterize cell physiology and signaling of β-cells *in vivo* using any of the ever-expanding spectrum of fluorescent protein biosensors. Despite the low percentage of β-cells expressing biosensor measured using flow cytometry, our study shows that the transduction of islets *in vivo* is not uniform and some islets highly express the biosensor. These highly labeled islets were selected for analysis of islet function.

Using the Grx1-roGFP2 biosensor, we demonstrated how this system can be used to characterize acute and chronic cytoplasmic ROS regulation, which may aid in determining why specific cell populations and subpopulations are more susceptible to ROS, even across species^30^. Though ROS is primarily generated in the mitochondria, literature supports the idea that the unregulated release of mitochondrial ROS into the cytosol and enhanced cytoplasmic production of ROS are key features in the progression of cellular oxidative stress^31,32^. Although used here for proof-of-principle studies, roGFP has the potential to be a highly relevant tool for diabetes research since evidence supports the conclusion that oxidative stress, and the subsequent triggering of β-cell apoptosis, is a common feature of both type 1 and 2 diabetes^2,33^. Using GcAMP6s, we further demonstrated how the platform can be rapidly modified to evaluate a different analyte and collected data demonstrating that calcium oscillations islets *in vivo* are similar to those measured *in vitro*^21^. GcAMP6s is an extremely relevant tool in islet biology since physiological regulation of insulin secretion critically depends upon tight regulation of intracellular calcium in β-cells, regulation that is disrupted in diabetes^34^. These studies offer a stepping stone to more complex analyses focused on specific signaling pathways, transgenic models, and disease states. With this in mind, the adeno- and AAV- applications were designed to permit the simple substitution of biosensors for other pathways of interest (e.g., LC3 for autophagy^35^ or AKAR4.1 for PKA signaling^36^) or even other promoter sequences to target other islet cell types. Furthermore, these virally-packaged biosensors can be utilized in virtually any mouse model without lengthy breeding of a biosensor transgene, and can also be used in human islets transplanted into immunodeficient mice.

While much can be learned from acute IVM studies, in which imaging of a surgically-exposed organ is only conducted once prior to sacrifice, additional information can be gleaned from longitudinal studies of the same living tissues conducted over time, particularly in the context of a progressive disease like diabetes. Studies of this kind typically require the use of an implanted window to enable repeated imaging of the same tissue without the need for repeated surgeries. Based upon a design developed and widely used by the van Rheenen group^26,37^, we applied an abdominal imaging window in tandem with AAV8-mediated gene delivery, to detect increases in ROS and concomitant decreases in β-cell volume in the MLD-STZ model of diabetes. It is known that STZ leads to β-cell death through ROS production^38^, which results in a concurrent increase in blood glucose levels and decrease in islet mass^27,28^. Our system allows us to not only recapitulate these observations, but also show that the loss in β-cell volume precedes the both rise of blood glucose and maximal ROS levels in individual mice. Our data provide unique support to the idea that the remaining β-cells that survived early STZ ablation are partially able to compensate for the loss in mass up to a “tipping point”^39^. While MLD-STZ is a widely used model for diabetes, the progression of diabetes in this model is imperfect. The flexibility of our system will make it possible to explore the role of different physiological factors and biochemical pathways in this and other, more targeted models of diabetes such as the *db/db* or non-obese diabetic (NOD) mice.

In conclusion, IVM is a valuable but underutilized tool for the study of islet-cell function *in vivo*^4,22^. Our highly-portable experimental platform, comprised of virally-packaged biosensor and AIW’s, facilitate targeted interrogation of β-cell biology in both transplanted islets and islets of the endogenous pancreas. The longitudinal IVM approach we demonstrate here thus provides a unique paradigm to study islet structure and cellular function within the context of steady-state drug treatments, β-cell replication or death, and diabetes progression.

## Methods

### End-point pancreas and islet transplant intravital imaging

All mouse experiments were performed with approval and oversight from the Indiana University Institutional Animal Care and Use Committee (IACUC). All experiments were performed in accordance with relevant guidelines and regulations. Male and female C57BL/6J mice, 8–12 weeks of age, were used for pancreas or kidney capsule islet transplant (methods below) imaging. On the day of imaging, the mouse was anesthetized with isoflurane and placed in the right lateral decubitus position and a small, 1 cm vertical incision was made along the left flank at the level of the pancreas or left kidney. The exposed organ was orientated so that the organ was underneath the animal, pressed gently on a 50 mm coverslip-bottomed dish (Pelco) for imaging on an inverted microscope. The animal’s temperature was maintained via a draped heating pad, heading pads for the stage, and heating elements for the objective to diminish the heat sink at the level of the objective tissue interface. A rectal thermometer was in place to keep a continuous read out on the mouse’s core body temperature. Intravenous access was achieved using the placement of a jugular catheter or by tail vein injection.

### Intravital reagents

To label the pancreas for IVM, several injectable dyes were used. Hoechst 33342 (1 mg/kg in sterile saline, Thermo) was used to label nuclei, a 150 k Dalton fluorescein-conjugated dextran (1 mg/kg in sterile saline, Sigma) and a 70 k Dalton tetramethylrhodamine dextran (1 mg/kg in sterile saline, Thermo) were used to label vasculature, and tetramethylrhodamine (TMRM, 5 ug/ml in sterile saline, Invitrogen) was used to label mitochondria. Alloxan monohydrate (Sigma) was resuspended in sterile saline at 80 mg/kg immediately prior to the IV injection and imaging. D-glucose (Sigma) was resuspended in sterile saline at 1 g/kg prior to IP injection.

### IVM microscope setup

All IVM-based confocal and multiphoton images were captured using a Leica SP8 (25x/0.95NA/Water immersion objective) outfitted with a MaiTai DeepSee (Spectra Physics) multiphoton laser. To image Grx1-roGFP2 *in vivo*, the biosensor was sequentially excited with 405/488 nm lasers at an 8:1 power ratio with emissions collected from 490–600 nm using internal detectors or with 800/900 nm lasers at a 1:1 power ratio with emissions collected from 500–575 nm using external detectors. To image GcAMP6s *in vivo*, the biosensor was excited with 920 nm excitation.

### Virus generation

Adeno- and adeno-associated viruses (AAV) were generated by VectorBuilder (Cyagen Biosciences). Briefly, the custom β-cell specific promoter contained 414 base pairs of the rat insulin-1 promoter, along with 691 basepairs of the rabbit beta-globin intron^11^ (sequence available in supplemental material). This promoter and Grx1-roGFP2 and GcAMP6s sequences were de novo synthesized and cloned into the necessary adeno- or AAV- expression vectors for viral production.

### *In Vivo* Islet Infection

To infect β-cells *in vivo*, AAV8-INS-Grx1-roGFP2 was injected intraperitoneally 14–21 days prior to endpoint intravital imaging, 7–28 days prior to flow-based expression studies, or 8 days prior to longitudinal AIW studies. A mouse was given a single dose of 2–2.5 × 10^11^ genome copies in 100 µl of saline.

### Flow cytometry for biosensor expressing β-cells

Isolated islets from mice dosed with AAV8-INS-Grx1-roGFP2 were dispersed using 1 ml of Accumax (StemCell Technologies) containing 2 U/mL of DNAse I for 10 min at 37 °C. Single cells were fixed and stained with anti-insulin (1/10 dilution, Dako) for 90 min at room temperature. Cells were washed and incubated with wash buffer containing secondary antibody (Alexa-fluor 568, 1/500 dilution, Invitrogen) for 30 min, room temperature. After the staining, the cells were washed, filtered and acquired on a FACS LSR II cytometer (BD). The cells were analyzed using FlowJo software (v 7.6.5, Tree Star). Initially, the cells were gated on FSC-A and FSC-H for doublet exclusion, followed by a strategy to gate on FSC (size) and SSC (granularity) to select a population compatible with β cells, within which the insulin-positive cells were evaluated, and finally GFP positivity of insulin-positive cells.

### Islet isolation

Male and female C57BL/6J mice 8 weeks of age (Jackson Labs) were utilized in both *in vitro* assays and islet transplant studies. 20 week-old female mice expressing an insulin-promoter driven ROSA26 nuclear H2B-mCherry^40^ were utilized for mCherry colocalization studies. To isolate islets, mice were euthanized, the pancreas was harvested, and islets were liberated using a 0.3% collagenase digestion in 37 °C shaking water bath in Hanks buffered sodium salt. Islets were maintained in islet media containing phenol free RPMI 1640, with 10% FBS, 100 U/ml Penicillin, 100 ug/ml Streptomycin, and 8 mM glucose. Islets were allowed to recover over-night prior to subsequent experiments.

### *In Vitro* islet infection, treatment and imaging

To infect β-cells *in vitro*, isolated islets were washed with Dulbecco’s phosphate buffered saline without calcium or magnesium, followed by distention with Accutase (Sigma) at 37 °C for 30 seconds. Accutase was rapidly inactivated with room temperature islet media, then washed with fresh islet media. Adenovirus was added directly to islet media to achieve approximately 2 × 10^7^ viral particles per 100 islets. Viral infection lasted at least 6 hours prior to image or transplantation the following day. After viral infection, islets were placed in fresh islet media with varying glucose concentrations (2, 8, 16.7, and 25 mM) for either 4 or 16 (overnight) hours. Islets were imaged *in vitro* using a Zeiss LSM 800 (40x/1.2 NA/Water Objective) equipped with an Ibidi Stage Top Incubation system (5% CO2, 37 °C, 85% Humidity). Grx1-roGFP2 was sequentially excited with 405 and 488 nm lasers at a 3:1 power ratio, with emissions collected from 490–600 nm. Alloxan monohydrate (Sigma) was resuspended in islet media at 4 mM immediately prior to the start of imaging.

### Islet transplants

Adeno-infected islets were transplanted into anesthetized recipient mice via sub-renal capsular injection under aseptic conditions. Mice were anesthetized with isoflurane. The left lumbar region received a single incision to expose the kidney. A small entry hole (~1 mm diameter) was made in the kidney capsule with a jeweler forceps and islets were gently released into the subcapsular space through a small piece of sterile polyethylene tubing attached to a syringe. The renal capsule was allowed to close by secondary intention, the body wall and skin incision were closed using monofilament 4.0 silk sutures, and the mice were allowed to recover. Analgesics were administered for pain relief. The mice were singly housed post-surgery. Islet transplants were allowed to engraft 7–14 days prior to intravital imaging.

### Abdominal imaging window (AIW) procedure

A general abdominal imaging window (AIW) procedure has been published previously^26^. Briefly, the window is constructed from surgical grade titanium to prevent rejection and consists of 2 parts: an outer ring with a deep groove to hold and hid the skin, peritoneum, and sutures and a removable inner ring that holds the glass coverslip. We adapted the surgical protocol to be successful over the pancreas. The animals were anesthetized via isoflurane and all procedures were performed under aseptic conditions. Warm sterile saline (1 ml) was injected subcutaneously to aid in fluid replacement. A vertical left flank incision was performed approximately 1.5 cm in length. The pancreas was identified, and externalized utilizing cotton tipped applicators. A purse-string suture (non-absorbable monofilament) was placed around the incision through both the fascial and the skin layers. Each suture was approximately 0.5 cm apart from the next and at least 0.1 cm from the edge of the incision. Cyanoacrylate adhesive was applied to the interior of the outer AIW ring and then it was placed on the pancreas. Mild pressure was applied for approximately 5 minutes to ensure that the ring adhered to the pancreas. The inner ring with attached coverslip was then placed into the outer ring and contact with the pancreas was visually confirmed. The skin and fascial edges were then placed into the groove of the AIW and the purse string suture was secured with at least 4 square knots. This effectively closed off the abdominal cavity and left the organ of interest in contact with the coverslip. Once the AIW was in place the mouse was allowed to emerge from anesthesia and placed in a recovery cage on a warming blanket with soft bedding. Analgesics were administered for pain relief.

### Intravital for AIW imaging

Mice with AIW’s were anesthetized with isoflurane for no longer than 30 minutes for imaging of labeled islets. Mice received 200 uL of sterile saline to prevent dehydration. STZ (Sigma)  injections were administered in the afternoon, while the imaging was completed in the morning to avoid measuring any acute effects of the drug, but instead to measure the chronic effects on ROS that persist over time. Z-stacks were sequentially collected using 800 and 900 nm excitation (1:1 power ratio) and a green emission filter (520–580 nm). At the end of imaging, duplicated blood glucose readings from a tail nick were measured using an AlphaTrack2 glucometer and test strips. Mice were allowed to recover prior to returning to animal housing.

### Data analysis, image processing and presentation

Background subtracted, raw images were analyzed using ImageJ (NIH) or Cell Profiler^41^. Intensity of the 405 nm-based emission was divided by 488 nm-based emission to create a raw Grx1-roGFP2 ratio. Ratiometric images were created using Cell Profiler and rescaled to an arbitrary linear look-up table that fit all data points within that experimental data set. The Cell Profiler Pipeline script is available in the supplemental data. To determine islet volume, an average intensity projection was generated from a z-stack of the imaged islet and the GFP-positive voxel area was calculated within the region of interest encompassing the baseline islet volume using ImageJ. Single plane slices within an islet z-stack that had significant motion artifacts from respiration were removed prior to the creation of the projection. Images presented in figures were linearly scaled for display purposes only. For statistical testing, Student’s t-tests and 1-way ANOVAs were used.

## Supplementary information

Supplementary Information

GcAMP6s Activity Video

## Data Availability

The authors declare that all data supporting the findings of this study are available within the paper and its supplementary information files.
