# Supplementary material for: A Versatile, Portable Intravital Microscopy Platform for Studying Beta-cell Biology In Vivo
Source: Sci Rep. 2019 Jun 11;9:8449. doi: 10.1038/s41598-019-44777-0 (PMC6559992; doi:10.1038/s41598-019-44777-0)
Supplement: Supplementary file 1 — Supplementary Information [file 41598_2019_44777_MOESM1_ESM.pdf]

Title: A Versatile, Portable Intravital Microscopy Platform for Studying Beta-cell Biology *In Vivo*

Authors: Christopher A. Reissaus[1], Annie R. Piñeros[1], Ashley N. Twigg[2], Kara S. Orr[1], Abass M. Conteh[4], Michelle M. Martinez[5], Malgorzata M. Kamocka[5], Richard N. Day[3,6], Sarah A. Tersey[1,2,3], Raghavendra G. Mirmira [1,2,3,4,6], Kenneth W. Dunn[3,5], Amelia K. Linnemann[1,2,3,4,6*]

Affiliations: [1]Department of Pediatrics, Indiana University School of Medicine, Indianapolis, IN, USA, [2]Herman B Wells Center for Pediatric Research, Indianapolis, IN, USA, [3]The Center for Diabetes and Metabolic Diseases, Indiana University School of Medicine, Indianapolis, IN, USA, [4]Department of Biochemistry and Molecular Biology, Indiana University School of Medicine, Indianapolis, IN, USA, [5]Department of Medicine, Division of Nephrology, Indiana University School of Medicine, Indianapolis, IN, USA, [6]Department of Cellular and Integrative Physiology, Indiana University School of Medicine, Indianapolis, IN, USA

RIP-1 – Beta Globin Promoter (1105bp)

gctgagctaagaatccagctatcaatagaaactatgaaacagttccagggacaaagataccaggtccccaacaactgcaa
ctttctgggaaatgaggtggaaaatgctcagccaaggaaaaagagggccttaccctctctgggacaatgattgtgctgtga
actgcttcatcaggccatctggccccttgttaataatctaattaccctaggtctaagtagagttgttgacgtccaatgagcgcttt
ctgcagacttagcactaggcaagtgtttggaaattacagcttcagcccctctcgccatctgcctacctacccctcctagagcc
cttaatgggccaaacggcaaagtccagggggcagagaggaggtgctttggactataaagctagtggagacccagtaact
cccaagctagcttcgaattctgcagtcgagctccaccgcggtggcggccgctctagtggatcctgagaacttcagggtgagtttgggg
acccttgattgttctttctttttcgctattgtaaaattcatgttatatggagggggcaaagttttcagggtgttgtttagaatgggaagatgtccct
tgtatcaccatggaccctcatgataattttgtttctttcactttctactctgttgacaaccattgtctcctcttattttcttttcattttctgtaactttttcgt
taaactttagcttgcatttgtaacgaatttttaaattcacttttgtttatttgtcagattgtaagtactttctctaatcacttttttttcaaggcaatcag
ggtatattatattgtacttcagcacagttttaggaacaattgttataattaaatgataaggtagaatatttctgcatataaattctggctggcgt
ggaaatattcttattggtagaaacaactacaccctggtcatcatcctgcctttctctttatggttacaatgatatacactgtttgagatgagga
taaaatactctgagtccaaaccgggcccctctgctaaccatgttcatgccttcttctctttcctacagctcctgggcaacgtgctggttgttgt
gctgtctcatcattttggcaaa

Supplemental Figure 1

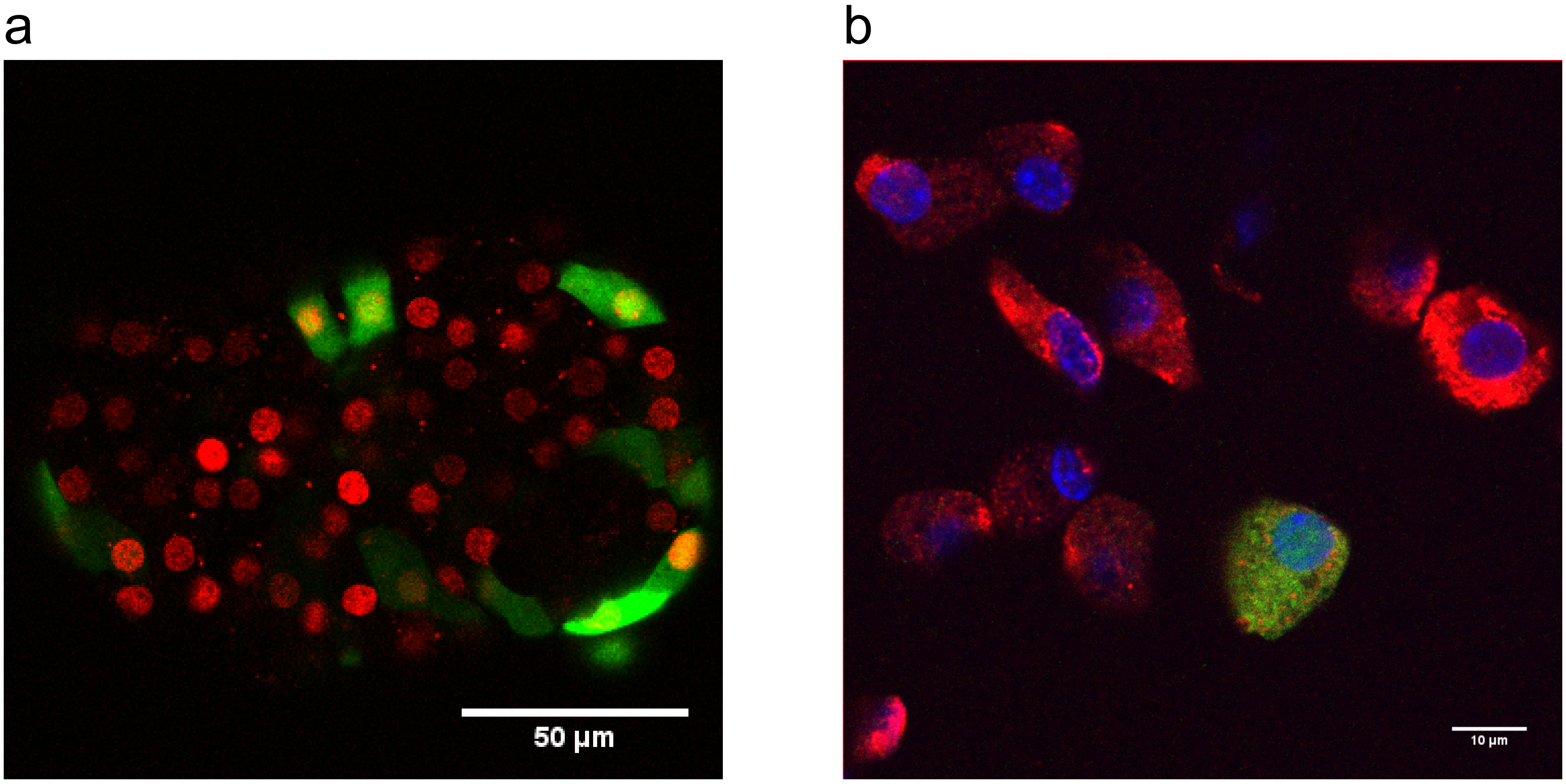

Figure S1: Colocalization of Adeno- and AAV8- INS-Grx1-roGFP2 *in vitro* and *in vivo*

(a) Isolated islets from mice expressing an insulin-promoter driven ROSA26 nuclear H2B-mCherry were infected with Adeno-INS-Grx1-roGFP2 and imaged using a Zeiss LSM 800 confocal (Green – Grx1-roGFP2, Red – mCherry). While not all β-cells are infected and express biosensor, all cells that are expressing roGFP2 are mCherry positive. (b) Islets were isolated from WT mice IP injected with AAV8-INS-Grx1-roGFP2 3 weeks prior and were dispersed and stained with an anti-GFP (to stain Grx1-roGFP2) and anti-insulin antibodies to determine the localization of Grx1-roGFP2 expression and imaged. While AAV8-INS-Grx1-roGFP2 does not label all insulin-positive cells, all GFP-positive cells are also insulin positive.

Supplemental Figure 2

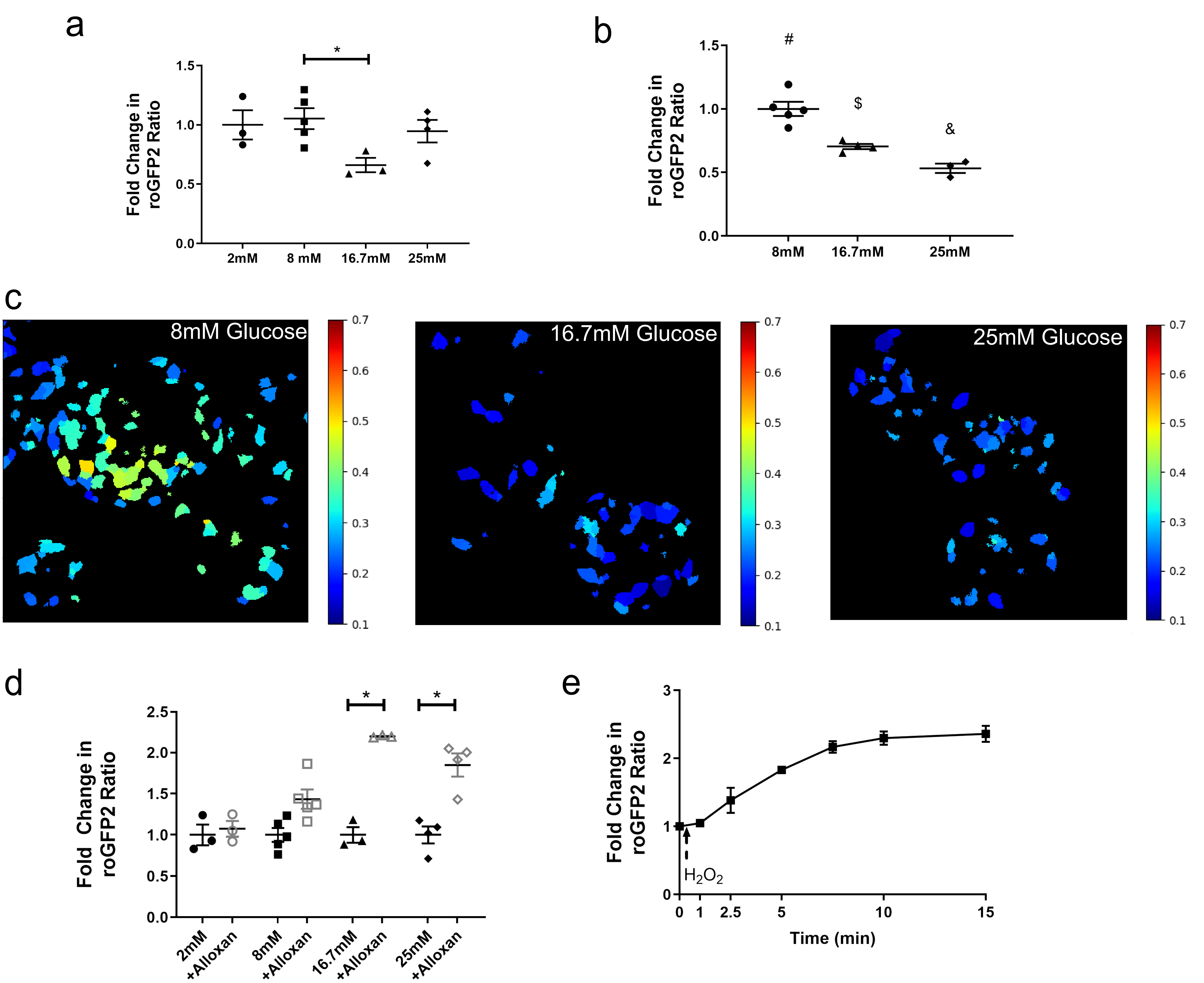

Figure S2: *In Vitro* Characterization of Adeno-INS-Grx1-roGFP2
(a) Adeno-INS-Grx1-roGFP2 infected primary islets from C57Bl/6J mice were incubated for 4 hours with islet media containing 2, 8, 16.7, or 25 mM glucose. Emissions (490-600nm) were collected from sequential 405 and 488 nm excitation. (b) Similar to a, except islets were incubated for 16 hours with islet media containing 2, 8, 16.7, or 25 mM glucose then imaged. (c) Representative ratiometric images generated from islets after 16 hours glucose incubations. (d) Adeno-INS-Grx1-roGFP2 infected mouse islets incubated for 4 hours with islet media containing 2, 8, 16.7, or 25 mM glucose (filled black) were then imaged after 5 minutes of stimulation with 4mM alloxan monohydrate (hollow grey). Data are plotted as a fold change relative to the baseline ratio for each mouse. (e) Similar to a, except Grx1-roGFP2 response was measured as the ratio of two-photon fluorescence excited at 800 nm to that excited at 900 nm over time after addition of 3uM H2O2. Data are means ±SEM. (N ≥ 3 mice; >4 islets per mouse; *p<0.05).

Supplemental Figure 3

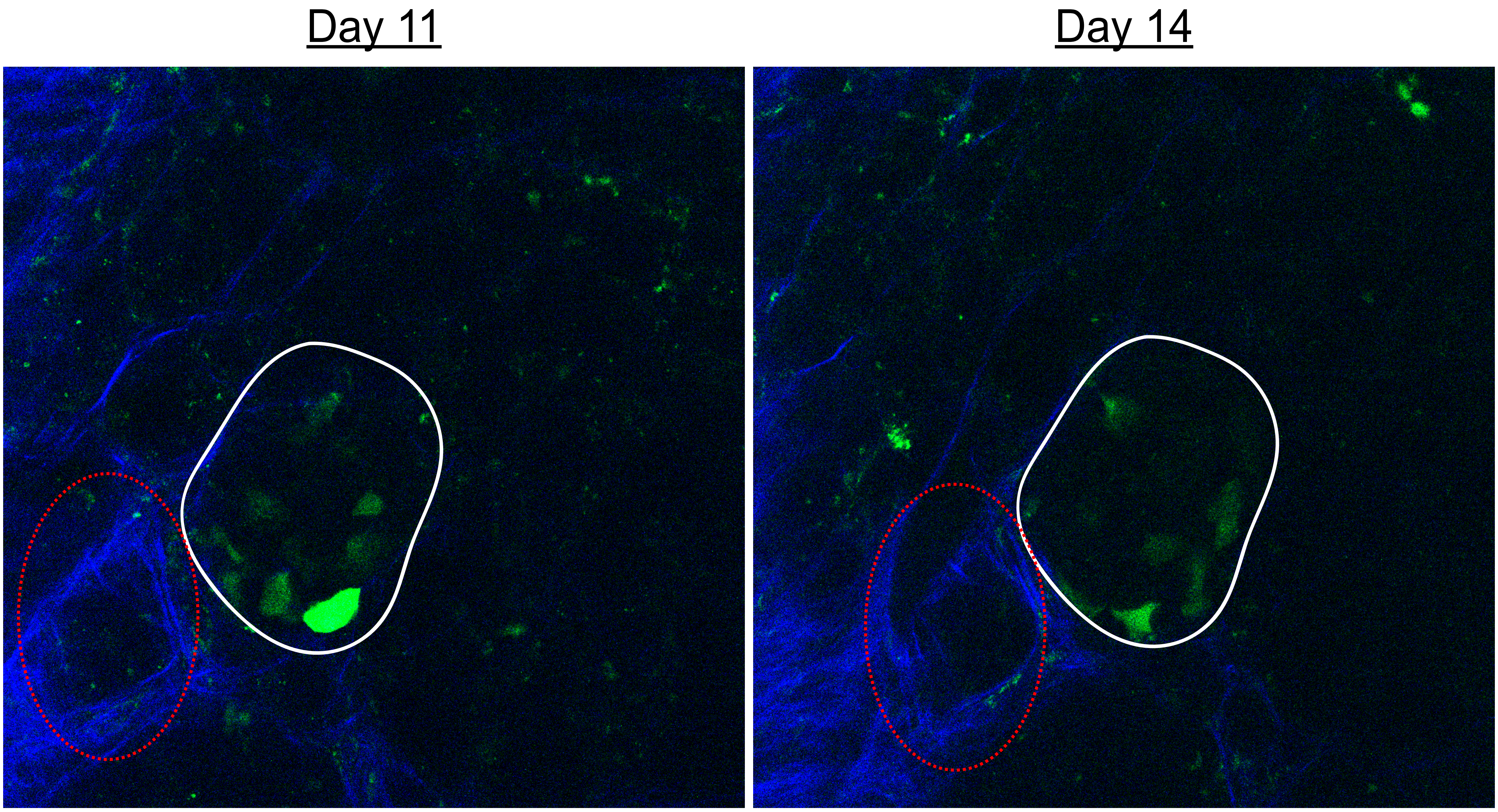

Figure S3: Second Harmonics Act as a Fiduciary Marker
Second harmonic generation from collagen was used as a fiduciary marker to return to islets between imaging sessions. Between days 11 and 14, the islet of interest is highlighted in white, while a unique collagen structure is highlighted in red.

Supplemental Figure 4

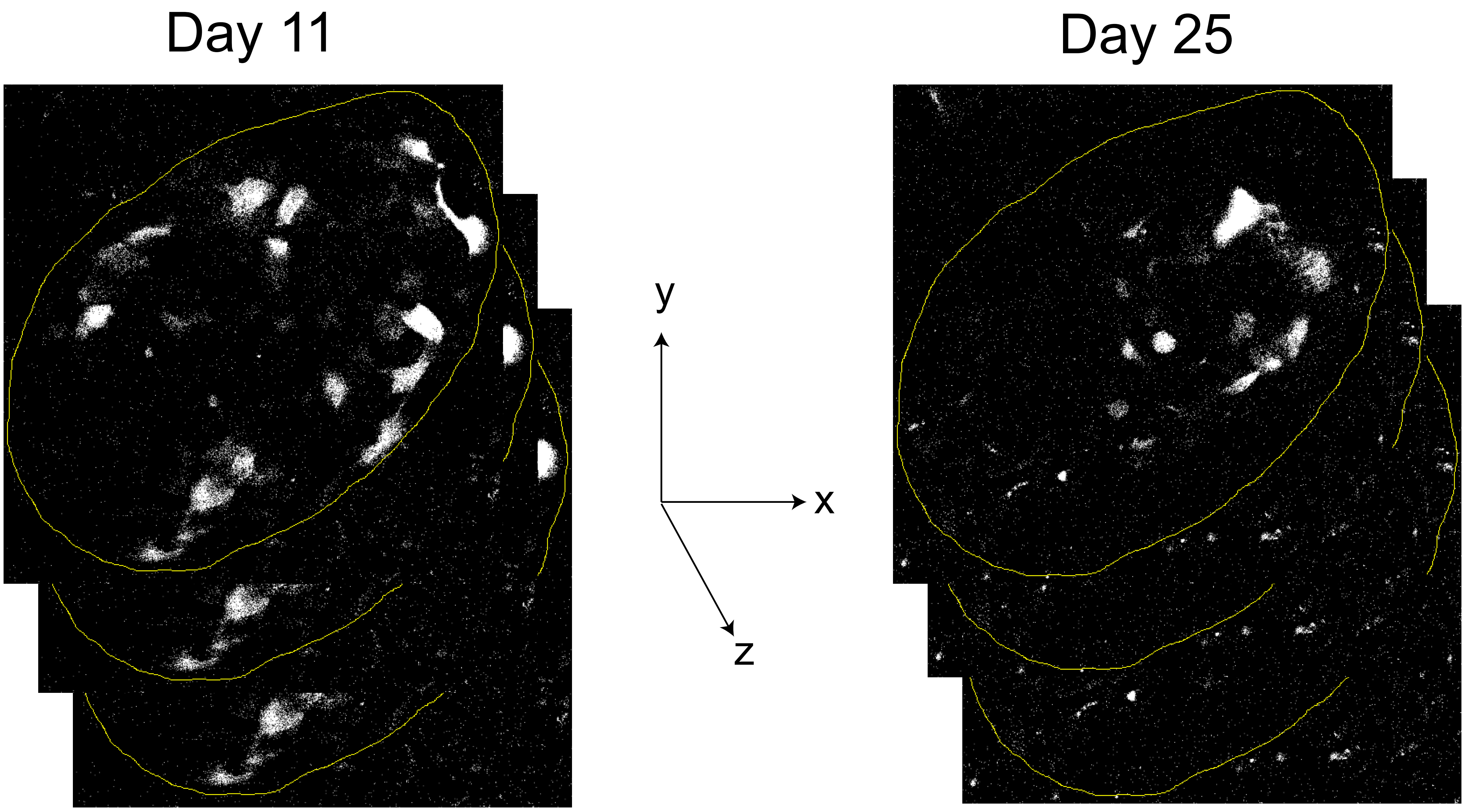

Figure S4: Measuring β-cell Volume in Longitudinal AIW Studies
To measure β-cell volume in the same islet over time, average intensity projections of the islet at each time point were generated, then the voxel area of GFP positive voxels was collected from within the islet area (yellow line) established at Day 11/Baseline. Thresholding parameters used to make the GFP-voxel mask were constant across each day for the same islet.
